# Effect of immunodeficiency and tumor marker expression on HIV-related diffuse large B-cell lymphoma prognosis

**DOI:** 10.1186/1750-9378-7-S1-P47

**Published:** 2012-04-19

**Authors:** Michael J Silverberg, Jonathan Said, Hongbin D Zha, Donald I Abrams, Otoniel Martinez-Maza, Michelle McGuire, Reina Haque, Margaret Chi, Lanfang Xu, Brandon Castor, Chun Chao

**Affiliations:** 1 Kaiser Permanente Northern California, Oakland, CA, USA; 2 University of California, Los Angeles School of Medicine, Los Angeles, CA, USA; 3 Kaiser Permanente Southern California, Los Angeles, CA, USA; 4 San Francisco General Hospital, San Francisco, CA, USA

## Background

Several tumor markers may predict survival in HIV+ patients with diffuse large B-cell lymphoma (DLBCL). Here, we evaluate the association of immunodeficiency (CD4<200) on expression of prognostic tumor markers and survival.

## Methods

HIV+ DLBCL cases diagnosed between 1996-2007 within Kaiser Permanente California were identified. H&E slides were reviewed to identify representative tumor blocks for tissue microarray (TMA) construction. Immunohistochemistry staining of TMA cores was used to detect the expression of selected markers in the categories of (1) cell cycle regulators, (2) B-cell activators, (3) anti-apoptotic proteins, and (4) others, including Epstein Barr Virus (EBV). Percent of DLBCL cells with visible marker staining was scored on a scale from 0-4 (i.e., 0-9%, 10-24%, 25-49%, 50-74% and ≥75%). EBV infection was determined by in situ hybridization of EBV RNA. We also considered high vs. low expression levels based on previously established cut-offs. Of the 20 markers previously examined, three had emerged as significant predictors of survival, including EBV, cMYC and BLIMP1. Here, we evaluated the association between CD4 and expression of these three markers by t-test for mean levels and chi-square for % high levels. We also evaluated the combined effect of immunodeficiency and marker expression on 2-year survival in unadjusted Cox models.

## Results

We identified 194 HIV+ DLBCL cases; 80 patients had adequate tissue for the marker analyses. Of the three markers, only EBV was associated with CD4 level (Table 1).

**Table 1:** Tumor marker levels by CD4

	EBV			cMYC			BLIMP1		
	CD4			CD4			CD4		

	<200	≥200	P	<200	≥200	P	<200	≥200	P

Mean levels	1.9	0.6	0.009	1.6	1.7	0.90	0.3	0.2	0.65
% high levels	45.7	16.0	0.016	68.6	64.0	0.71	11.1	8.0	0.69

Survival was lowest in cases with high levels of EBV or cMYC in combination with low CD4 (Table 2). Survival was not evaluated for BLIMP1 given the low prevalence.

**Table 2:** Two-year survival by CD4 and marker levels

	EBV			cMYC		
	HR	95% CI	P	HR	95% CI	P

Low CD4/high marker	4.0	1.6-10.2	0.004	3.3	1.0-11.5	0.057
Low CD4/low marker	1.8	0.7-4.9	0.236	1.0	0.2-4.8	0.970
High CD4/high marker	1.6	0.3-7.7	0.557	1.3	0.3-5.1	0.739
High CD4/low marker (ref)	1.0	-	-	1.0	-	-

## Conclusion

Immunodeficiency was associated with EBV+ DLBCL. Cases with low CD4 and high levels of EBV or cMYC had worse survival. Risk stratification may consider both CD4 and tumor marker expression, although confirmation is needed in larger studies.

